# Canadian adolescent mothers’ perceptions of influences on breastfeeding decisions: a qualitative descriptive study

**DOI:** 10.1186/1471-2393-12-149

**Published:** 2012-12-12

**Authors:** Sherry A Nesbitt, Karen A Campbell, Susan M Jack, Heather Robinson, Kathleen Piehl, Janice C Bogdan

**Affiliations:** 1 Durham Regional Health Department, 605 Rossland Road East, Whitby, Ontario, Canada; 2 School of Nursing, McMaster University, 1280 Main Street West, Hamilton, Ontario, Canada

**Keywords:** Breastfeeding, Young mother, Adolescent, Teenage mothers, Breastfeeding support, Commitment, Barriers, Qualitative

## Abstract

**Background:**

There is increased recognition of the importance of breastfeeding at a national level as evidenced by the increased number of Canadian mothers initiating breastfeeding. However, adolescent mothers (<19 years), compared to all other mothers, have lower rates of breastfeeding initiation and duration. The purpose of this study was to examine the facilitating influences and barriers to initiating, and continuing breastfeeding, as perceived by adolescent mothers in Durham Region, Ontario, Canada.

**Methods:**

The principles of interpretive description guided this qualitative study. A purposeful, homogenous sample of 16 adolescent mothers (15–19 years) were recruited to complete individual, semi-structured, face-to-face interviews. Conventional content analysis was used to code data, identify concepts and synthesize them into overall themes.

**Results:**

Adolescent mothers in this study expressed that the decision to breastfeed was made prenatally and while partner and family member opinions about breastfeeding initiation were influential, the decision was made independently. Mothers were primarily motivated to initiate breastfeeding due to the health benefits for the infant. Lower breastfeeding duration rates were found among mothers who decided to only “try” breastfeeding when compared to the mothers who committed to breastfeeding. Influences on continued breastfeeding included: 1) the impact of breastfeeding on social and intimate relationships; 2) the availability of social support; 3) the physical demands of breastfeeding; 4) mothers’ knowledge of breastfeeding practices and benefits; and 5) mothers’ perceived sense of comfort in breastfeeding.

**Conclusions:**

The results of this study provide health care providers new conceptual insight and understanding of the factors that influence adolescents’ decisions to “try” breastfeeding and to continue providing breastmilk to their infants. Professional implications drawn from this study include active engagement of adolescents in the pre and postnatal periods, including early assessment of potential barriers surrounding breastfeeding decisions. This early professional interaction highlights the professional as a form of support, and allows for sharing of evidence-informed breastfeeding information and practical breastfeeding skills. Inclusion of adolescents’ positive social support networks should be emphasized in professional breastfeeding support. Motivational interviewing is a promising prenatal strategy to influence behavior change and reduce ambivalence in decision-making about breastfeeding, creating opportunities for health care providers to tailor interventions.

## Background

Internationally, breastfeeding is recognized as the optimal method to meet infants’ nutritional requirements [1,2]. Additionally, the consumption of human milk has significant health benefits in that it lowers an infant’s risk for multiple acute and chronic childhood diseases [1]. A comprehensive systematic review of the benefits of breastfeeding on maternal and infant health outcomes within developed countries, concluded for infants being breastfed, that there are associated reductions in the risks of: acute otitis media, non-specific gastroenteritis, severe lower tract respiratory infections, atopic dermatitis, asthma in young children, obesity, type 1 and 2 diabetes, childhood leukemia, Sudden Infant Death Syndrome and necrotizing enterocolitis [3]. Maternal health outcomes associated with breastfeeding included reductions in the risks of developing type 2 diabetes, and breast or ovarian cancers [3]. In addition, an association between not breastfeeding and post-partum depression has been identified [3]. The significant evidence base substantiating the benefits of breastfeeding on maternal and infant heath outcomes has resulted in organizations such as the World Health Organization [2], Health Canada [1], and the U.S. Department of Health and Human Services [4] recommending breastfeeding as the norm for infant feeding; with a focus on exclusive breastfeeding for the first six months followed by the subsequent introduction of complimentary foods and continued breastfeeding.

There is increased recognition of the importance of breastfeeding at a national level as evidenced by the rise in the number of Canadian mothers initiating breastfeeding. The Public Health Agency of Canada [5] cited that 85% of Canadian mothers in 2003 reported some breastfeeding compared to 75% in 1994–1995. A Canadian report in 2009 cited that approximately 90% of all new mothers initiated breastfeeding, however, when the national initiation rates are examined according to maternal age, adolescent mothers (<19 years of age) initiate breastfeeding less frequently (83.6%) compared to their older counterparts (88.5% - 92.7%) [6]. The greatest contrast when examining breastfeeding by maternal age can be seen in the rates of continued breastfeeding. At 6 months postpartum, 22% of adolescent mothers report any breastfeeding [6]. In comparison, 6 month duration rates among slightly older mothers (20–24 years) was higher at 37.1% and mothers older than 25 years were the most successful with continued breastfeeding to 6 months, with rates between 51.3% to 69.7% [6]. The multitude of risk factors facing adolescent mothers coupled with low breastfeeding rates highlights the importance of breastfeeding for this population.

The World Health Organization [2] states that breastfeeding reduces child mortality and contributes to the health of mothers. Adolescent motherhood is associated with numerous adverse health outcomes making the importance of breastfeeding for mother and infant crucial [7]. There is evidence that adolescents have higher rates of obstetrical complications and increased rates of low birth weight infants [7]. Infants born to young mothers are at increased risk of neglect and behavior concerns [8]. Another study suggests that this may be because adolescent mothers experiencing high prenatal or parenting stress are more likely to have emotional distress and low maternal adjustment [9]. While infants born to adolescent mothers are at higher risk for morbidity and mortality, breastmilk provides protection against a myriad of illnesses and diseases for the infant and promotes sensory and cognitive development [10,11]. Adolescent motherhood is associated with lower maternal educational attainment and earned income as well as increased rates of child poverty [12] however the benefits of breastfeeding may counteract some of the socioeconomic disadvantages facing this population [13].

There is a significant body of research that describes the factors associated with the initiation and continuation of breastfeeding among adolescent mothers. It has been identified that the pregnant woman’s mother and the infant’s father have the greatest levels of influence in her decision to breastfeed [13,14], as well as on the continuation or termination of breastfeeding. An adolescent in an intimate relationship with another teenager was more likely to breastfeed compared to a young mother with an older partner [15]. There is emerging evidence that formal types of support may also influence breastfeeding practices. It was found by Dennis [16] that primiparous mothers, over the age of 16, who participated in an antenatal peer support, were more likely to continue breastfeeding at 3 months and with greater exclusivity compared to mothers who were randomized to the conventional hospital and community supports. Wambach and Cohen [17] cite the impact that family and friends can have towards encouragement of breastfeeding, while also noting the importance of early support from professionals. This study also notes the impact of school systems as a positive or negative support. When the environment is supportive, it can be an enabling factor to breastfeeding [17]. Multiple studies have cited the embarrassment of exposing breasts as a factor that inhibits the initiation of breastfeeding among adolescent mothers [13,15,17-19].

Previous literature examining adolescents’ experiences with breastfeeding indicate that breastfeeding role models impact both adolescents’ attitudes towards breastfeeding as well as their own beliefs about infant feeding [14]. In a review of the literature, Wambach and Cole [13] found that adolescents exposed to breastfeeding role models were more likely to choose breastfeeding when faced with the decision of how to feed. Further substantiation of this came from another study that found a lack of breastfeeding role models may deter initiation [15].

Adolescents cite the significant infant health benefits as the rationale for choosing to breastfeed their baby [18,20], however many studies suggest adolescents lack knowledge of benefits, the practical skills to breastfeed [20-23], and hold misconceptions about breastfeeding [13]. In addition, deficits in breastfeeding knowledge and practical breastfeeding skills were reasons for breastfeeding cessation [23]. In a study by Wambach and Cohen [17], it was found that adolescents lacked information about milk supply. Avery, Zimmerman, Underwood and Magnus [22] cited reasons for not breastfeeding as: not enough milk, baby could not latch, and baby preferred formula. This same study identified a commitment to breastfeed was needed to overcome a lack of knowledge [22]. Feldman-Winter and Shaikh [24] found that education about the health benefits of breastfeeding may help adolescents to make a commitment to breastfeeding. Some literature has found that adolescent mothers’ knowledge and breastfeeding skills are associated with the confidence to breastfeed which increases competence in breastfeeding [14,25,26].

Previous literature provides direction for targeted support for breastfeeding adolescents to improve breastfeeding initiation and duration [23,27]. Additionally, there is some evidence to support integrating information about breastfeeding within school-based programs for pregnant adolescents to address their gaps in knowledge [23,24]. Other literature supports school exposure to breastfeeding to influence adolescents in their future decisions about breastfeeding [13,14].

Further investigation is needed, utilizing the perspectives of adolescents, to understand what breastfeeding supports are needed and who would be best to provide them. This current research compliments existing literature by providing insight into the breastfeeding experiences of Canadian adolescents in a regional area where breastfeeding rates are persistently low when compared to national statistics.

### Adolescent breastfeeding rates in Durham Region, Ontario

Durham Region is a large region characterized by rapid population growth; it is the largest geographical area in the Greater Toronto Area, made up of both urban and rural communities. It is located in Southern Ontario, Canada and has a population of over half a million. In 2007, the number of hospital deliveries for mothers aged 15 to 19 years old was 232 for Durham Region out of 5,256 Ontario births for this same age group [28].

In Durham Region, breastfeeding initiation and duration rates among adolescents fall below those of adult mothers. A comparison of breastfeeding rates by maternal age in Durham Region from 2006–2009, showed mothers, aged 15 to 19 had an initiation rate of 79% versus mothers over the age of 20 whom have an 89% initiation rate. Adolescent mothers in Durham Region were also significantly less likely to continue breastfeeding than adult mothers. Only 17% of adolescent mothers continued to breastfeed for at least 6 months compared to 49% of adult mothers [29]. This information highlights that Durham Region adolescent mothers are initiating and maintaining breastfeeding less than the national adolescent rates and far less often than older mothers.

This vulnerable population faces a constellation of health risks and consequently an understanding of adolescent mothers’ perspectives of breastfeeding is imperative for developing age appropriate interventions. Durham Region Health Department provides public health services to this large, diverse region within South-central Ontario. An overall goal of the Health Department, as guided by the Ontario Public Health Standards, is to improve breastfeeding rates among all mothers, with an additional focus on identified priority populations with lower breastfeeding rates. Examining the adolescent populations’ experiences of breastfeeding barriers and facilitating factors will allow for a deeper understanding of how public health practitioners can target interventions to improve maternal and infant health outcomes and reduce health inequities.

## Method

A qualitative approach was most appropriate for understanding and describing this phenomenon of adolescent mothers’ experiences of breastfeeding. The principles of interpretive description methodology [30] were applied to answer the research question, “How do adolescent mothers (15–19 years old) of infants describe and explain the factors that influence their decisions about their breastfeeding practices?” This qualitative research approach provides guidelines for describing and interpreting how individuals experience phenomena, particularly within the context of health and illness. Interpretive description is a qualitative methodology developed for addressing clinical questions within the applied health context [30]. It provides a flexible structure for inductively describing a phenomenon, or understanding it from the perspective of the individuals experiencing the phenomenon of interest. This description is then expanded to include an interpretive explanation that takes into consideration a range of influences, including culture, social, economic, political, or developmental ones [30]. The creation of these interpretive descriptions can then be used to effectively inform nursing knowledge and practice [31]. Approval to conduct this study was granted by the Durham Region Health Department Scientific and Ethical Review Committee.

### Setting and study participants

A purposeful sample of Durham Region adolescent mothers with infants (12 months of age or less) were sought to participate in this study. Participant inclusion criteria were as follows: 1) able to fluently speak English; 2) age between 15 and 19 years; 3) the biological mother of an infant less than or equal to 12 months of age; and 4) experience in breastfeeding to provide infant nutrition. Initiation of breastfeeding was defined as at least one attempt by the mother to feed the infant at breast. Given the homogeneity of the proposed sample, we estimated recruiting 15–20 participants or sampling until data saturation was achieved.

This sample of mothers was recruited via poster advertisements displayed in a variety of regional health and social care agencies that provide services to young pregnant women and parents. Interested teenage mothers who viewed the promotional poster were instructed to directly contact a member of the research team. Additionally, members of the research team (HR, KP) conducted two information sessions to outline the study objectives at a local community agency that provides educational programming for teenage parents. Following each session, mothers who were interested in participating in the study were encouraged to contact one of the researchers. All individuals who volunteered to participate and who were determined to have met the study inclusion criteria were interviewed.

### Data collection

To gain an in-depth description of the mothers’ breastfeeding practices and an understanding of the different factors influencing their decisions to continue breastfeeding, each participant completed a semi-structured, face-to-face interview. Table 1 details the guiding questions. During the interview, each participant was invited to describe and discuss her personal experience of breastfeeding her infant, to outline the decision-making process that informed her personal choice to breastfeed, to identify the facilitating factors and barriers that influenced her decision and ability to breastfeed, and similarly her decision to stop breastfeeding. The participants’ perceptions of the influence that their formal and informal support networks played on their breastfeeding decisions were also explored. Data were collected between June 2008 and January 2009. Each adolescent participant gave their informed consent to participate in the study; additionally each individual was informed that their participation was voluntary and that anonymity and confidentiality would be maintained.

**Table 1:** Questions guiding the interviews

1	Tell me about your breastfeeding experience.
2	All pregnant women are faced with the decision of how they are going to feed their baby. Can you tell me about how you made that decision?
3	Can you discuss the things that supported your attempt or experience with breastfeeding?
4	Can you discuss the things that made your attempt or your experience with breastfeeding difficult?
5	Some people say that social support (friends, family, neighbors, people you meet at school, work, and support groups) really affect your attempt or experience with breastfeeding. Can you tell me how the people around you affected your attempt or breastfeeding experience?
6	Some people say that support from professionals (doctors, nurses, midwives, counselors, lactation consultants, community groups and prenatal classes) can affect their attempt or breastfeeding experience. Can you tell me about your experience with any professionals or services?

To promote overall data credibility, two members of the research team (HR, KP) conducted the in-depth interviews. These nurse-researchers have extensive public health nursing experience working with adolescent mothers and vulnerable families in the context of delivering home visits, facilitating community-based pre- and post-natal programs for vulnerable families and teaching prenatal classes for pregnant adolescents. Within the scope of their professional nursing practice, these individuals were identified as having a strong foundation of knowledge related to breastfeeding, pre and post natal health, community supports and services, particularly with regard to high-risk families. This knowledge allowed them to probe for richer detail within the context of each interview. Furthermore, these nurses had the communication skills to quickly establish rapport, build trust and engage with the adolescent mothers who participated in this study; a necessary set of skills required to obtain a depth of data relevant for a qualitative study.

Participants were informed that the objective of the study was to describe and understand adolescents’ experiences of breastfeeding. The public health nurses who conducted the interviews were not known to the study participants, however participants were aware of the interviewers’ professional status as a nurse who works within the field of maternal-child health. The interviews were conducted in a location of the participant’s choice; in general, interviews were thus conducted in the participant’s home or in a private room in her school. Permission to tape-record each interview was obtained and the length of the interviews ranged from 25 to 80 minutes. The interviewer maintained field notes during the interview to document the major themes that emerged from the mothers’ descriptions of their breastfeeding experiences, including the barriers and facilitators to initiating and maintaining breastfeeding. At the end of each interview, these themes were reviewed with the participant as an initial member checking strategy to enhance overall data credibility. Participants also each completed a short demographic questionnaire. As an honorarium for her time and participation, each adolescent mother was provided with a $10 grocery voucher at the end of the completed interview.

### Data analysis

Data collection and analysis was conducted concurrently so that as new concepts emerged across interviews they could be explored in-depth in the remaining interviews. All interview data were transcribed verbatim and the qualitative software program N Vivo 8.0 was used to manage, code and sort data. Hsieh and Shannon’s process of conventional content analysis was used to code the data, identify concepts, and synthesize them into overall themes [32]. From among three variations of content analysis described [32], conventional content analysis is identified as an analytic strategy appropriate for descriptive studies, where there are few theoretical models available to describe the phenomenon of interest. This approach is further characterized by inductive coding where codes and categories emerge from the data rather than being determined a priori by the researcher. Once data were transcribed, members of the research team independently read each transcript and wrote a brief summary of the significant statements and concepts contained with the data. Following this initial step, members of the research team met to discuss the emerging concepts and through consensus, an initial coding scheme of the predominant themes was developed. This coding scheme was then used to consistently code all of the interview transcripts. Small clusters of codes were then collapsed into broader, more meaningful categories [32]. Important quotes illustrating the primary concept within each category were identified within the data [32]. In the last stages of analysis, core members of the research team (KC, SN) met again to review the data captured under each code and synthesized the concepts to create a rich description of the participants’ experiences. The team responsible for the initial coding analyzed the data through the lens of experienced public health nurses and a nursing manager with multiple years of experience working with breastfeeding mothers and vulnerable families, including pregnant and parenting adolescents. In interpretive description, professional experiential knowledge is valued as a strategy for “scaffolding” the study or establishing the base of evidence and experiences upon which the study will build [30]. Finally, node reports of each significant code, as well as the written synthesis of the concepts were shared with an experienced qualitative nurse-researcher (SJ) who has content expertise in public health interventions, maternal-child health and adolescent mothers for the purpose of conducting an audit of the codes in order to promote overall data dependability.

## Results

Sixteen adolescents from southern Ontario participated in this study to explore the enabling factors and barriers to breastfeeding. The average age of the study participants was 18 years (range from 17 to 19 years). The majority of participants identified grade 11 as highest education level achieved. All of the participants disclosed a low income, the majority ranged between $601.00 and $1000.00 per month. All of the participants were first-time mothers (first live birth) and at the time of the interview, the average age of the infants was 6 months, with a range of 3 to 11 months. A summary of the characteristics of each individual participant is provided in Table 2.

**Table 2:** Participant characteristics

**ID**	**Maternal Age**	**Infant Age**	**Breastfeeding Duration**	**Monthly Net Income**	**Highest Education Level Completed**
**01**	17	8 months	2 months	Did not answer	Grade 11
**02**	19	11 months	2 months	Less than $600	Grade 10
**03**	19	6 months	6 months (ongoing)	$601 - $1000	Grade 11
**04**	18	8 months	2 days	$601 - $1000	Grade 9
**05**	19	4 months	6 weeks	$601 - $1000	Grade 11
**06**	18	3 months	4 weeks	$601 - $1000	Grade 11
**07**	18	6 months	4 months	$0 - $600	Grade 9
**08**	19	6 months	ongoing	$1301 - $1600	Grade 12
**09**	17	3 months	5 weeks	Less than $600	Grade 9
**10**	19	4 months	6 weeks	$601 - $1000	Grade 10
**11**	18	8 months	2 days	$601 - $1000	Grade 11
**12**	18	6.5 months	3 weeks	$601 - $1000	Grade 10
**13**	18	3 months	2 weeks	$601 - $1000	Grade 11
**14**	17	4 months	4 months ongoing	No income	Grade 11
**15**	17	5 months	3 months	$601 - $1000	Grade 11
**16**	17	11 months	11 months ongoing	Did not answer	Grade 11

Note: Demographic information was collected at the time of the interviews.

Purposeful recruitment of young mothers occurred, which included those whom initiated breastfeeding, regardless of exclusivity or duration. At the time of the interview, 4 mothers were breastfeeding and 12 mothers had discontinued breastfeeding. The introduction of formula ranged from the infants’ first day of life to two months, and for three participants no formula had been introduced. Of the 12 adolescents who had discontinued breastfeeding, duration ranged from 2 days to 4 months.

The majority of adolescent mothers who participated in the study expressed that the decision to initiate breastfeeding was made during the prenatal period and while the opinions of their partner and family were influential, this decision was made independently by the mother. Participants emphasized the benefits of breastmilk on infant health as a primary motivator for deciding to breastfeed. Mothers who decided to only “try” breastfeeding had lower duration rates compared to the mothers who committed to breastfeeding their infant. In the postpartum period, multiple factors were identified that influenced the maternal decision to continue breastfeeding, including: 1) the impact of breastfeeding on social and intimate relationships; 2) the availability of social support; 3) the physical demands of breastfeeding; and 4) mothers’ perceived sense of comfort in breastfeeding. Across the mothers’ descriptions of their breastfeeding experiences, what emerged from the narratives was a lack of knowledge about breastfeeding norms and practices.

### Making the decision to breastfeed

The decision to breastfeed was made prenatally by the majority of the mothers. As one mother (Participant 8) said, “When I first got pregnant, I said ‘I want to breastfeed, I hope I can breastfeed.’ I said, ‘even if it’s hard, I still want to try and fight so I at least feed him for the first few months.” Only one participant indicated the decision was made postpartum while still in the hospital following the infant’s birth.

The adolescent mothers indicated that while their partners and family members’ opinions about breastfeeding were important to consider, the majority (n = 15) confirmed that they felt ownership over this personal decision. As one mother (Participant 1) declared, “I made the decision [for my baby], so it does not matter what everybody thinks.” Only one participant identified, that while the ultimate decision to breastfeed was hers alone, she was strongly influenced by her partner who had “told her to do it [breastfeed].” Most of the mothers described receiving extensive encouragement from their partners and family members to breastfeed. One young mother (Participant 9) expressed:

"I was completely encouraged by my family as well. I knew I was going to breastfeed, I was going to try my hardest, but my family was like, ‘You have to do it,’ you know, they really wanted me too."

Without prompting, four of the study participants specifically spoke to the high levels of support and encouragement they had received from male members of their family (e.g. their partner or father) to initiate breastfeeding. Overall, the breastfeeding norms and past experiences within the family context served to influence the adolescent when making the decision to breastfeed.

Study participants identified their decision to breastfeed was strongly influenced by having a close female family member with a previous positive breastfeeding experience. These female family members were also identified as key sources of support for the new mother. Some study participants also shared that family members who had formula-fed their infants or who experienced difficulties in breastfeeding focused on sharing their negative experiences or even projected that the participant would experience the same challenges. For example, one participant (5), shared that her mother had experienced breastfeeding challenges because “her milk supply just didn’t come in at all” and thus her mother told her that she “would probably be the same.” For several participants in this study, despite hearing about potential barriers and negative breastfeeding experiences, they still chose to breastfeed.

The reasons for choosing to breastfeed were also explored with the adolescent mothers in this study. The primary reason given was the benefits of breastmilk for their infant. Secondly, the mothers identified the economic benefits of breastfeeding as a motivating factor. Finally, mothers perceived that breastfeeding their infants would promote bonding. The mothers did not identify factors related to maternal health benefits as rationale for the decision to breastfeed.

### Commitment to breastfeeding

Many participants identified that they were committed to “try breastfeeding.” What emerged from the interviews were two categories of mothers, 1) those willing to “try” breastfeeding out of a sense of obligation or 2) those determined to “try” despite feelings of apprehension about the pain or difficulties associated with the process. For the former group, the mothers felt obligated to not “let down” their family members or partners who were encouraging them to breastfeed. These mothers were also keenly aware of the messaging communicating the benefits of breastfeeding and felt they had a responsibility to give their baby the best start in life. One mother (Participant 12) discusses the influence of these different factors on her commitment:

"As for my family, I kind of felt obligated to [breastfeed] in the beginning because my mom and all my sisters breastfed for as long as they could. So I had to try… Plus, the government and all those commercials you see on TV for breastfeeding your baby, it kind of makes you feel like you have to [breastfeed], even though you really don’t [have to]."

Furthermore, mothers who expressed an early low level of commitment to breastfeeding had duration rates ranging from 2 to 60 days.

In comparison, the latter group disclosed that some friends and family tried to discourage the process of breastfeeding through the sharing of their own negative personal experiences. As one mother (Participant 10) explained, “My friends discouraged me because they said it was going to hurt…I still said I would give it a try.” Of the participants who were breastfeeding at the time of the interviews, a commitment to breastfeeding was found to have a strong impact on breastfeeding duration; among these mothers, the breastfeeding duration rate ranged from 4 to 11 months. A strong relationship between the expectations they had of themselves to breastfeed helped overcome barriers that arose. Each of these mothers expressed commitment to breastfeed rather than to just “try.” They made comments such as, “When I found out I was pregnant … I decided to breastfeed … you have to be committed to it and it’s really hard” (Participant 9) and

"I knew one [friend] she went up to a year and that was my goal I wanted to go at least a year. And then she made a year and I was like, “hmmm if she can do it I can do it.” (Participant 7)"

### Factors influencing maternal continuation of breastfeeding

#### Impact on social and intimate relationships

Socially, one-quarter of the study participants disclosed that they found breastfeeding to be very convenient and that it facilitated their ability to engage in social activities outside of the home. Participants spoke specifically to the convenience of not having to clean bottles or prepare formula. However, more than half of the mothers in the study discussed the negative impact that breastfeeding routines and practices had on their ability to engage socially with their friends or to maintain their intimate relationships with their partners. The mothers valued “going out” and many of them expressed that breastfeeding left them feeling “trapped” or “stuck” and that “you can’t go out and do the things you need to do.” Some mothers also felt left out of social events and identified that breastfeeding limited their ability to participate in the types of activities that they engaged in pre-pregnancy. For many of these mothers, breastfeeding further contributed to their sense of social isolation because of their discomfort in feeding in front of other people or in public places. As one mother (Participant 12) expressed, “[It] kind of sucked…sitting in the bedroom for an hour or 45 minutes, just all by myself.” As young mothers, the study participants also explained that they felt judged by others in society and that breastfeeding in public created another opportunity to be scrutinized. One mother (Participant 5) explained, “I felt like I didn’t really want to go anywhere… Well it’s just that as a young mother people are watching you, and then breastfeeding on top of it, it was just really hard.” The ability to give the infant a bottle allowed these mothers time away from the infant, feel comfortable feeding in front of friends and to continue to engage socially.

A few mothers in this study discussed the impact of breastfeeding on their intimate partner relationships. For most of these mothers, partners were supportive of their breastfeeding and even identified ways to assist in the process. One participant (9) shared, “My boyfriend was just helping…He’d bring me the baby and when I was sitting down he’d bring me a drink or a snack.” Only one mother in the study identified that breastfeeding was perceived as interfering with the quality of her intimate relationship with her partner and the amount of time they could spend together. This mother highlighted the importance of the intimate relationship and the ease that bottle-feeding afforded her so that “somebody else can take [my son]” (Participant 1). For many mothers in the study, bottle-feeding could allow them to spend their free time within their peer and intimate relationships, illustrating the impact parenthood and breastfeeding has on their abilities to meet the milestones commonly associated with adolescent development.

#### Physical demands of breastfeeding

For the majority of the mothers in this study, the physical demands of breastfeeding were identified as a significant factor that influenced their ability and desire to continue breastfeeding. Over half of the mothers described that breastfeeding was more difficult than they expected. There was also the perception that it took a lot of time during the day to breastfeed and that breastfed infants needed to feed more frequently compared to babies receiving formula. Most of the participants explained that they felt tired and exhausted while breastfeeding and held the primary responsibility to wake up at night to breastfeed. With minimal nighttime help, their sleep patterns were interrupted and they were constantly tired. As one mother (Participant 7) expressed, “It feels like you are a 24 hour food bank.” As exhaustion and the physical demands of breastfeeding increased, some of the mothers experimented with supplementation by bottle. The experience of offering a bottle was perceived positively as their infants required shorter and less frequent feeds; the responsibility of feeding could be handed over to another individual; and there was an end to the discomfort and perceived inconveniences of breastfeeding. The same mother (participant 7) further stated, “Sometimes, the bottle is your saviour.” Following the introduction of a bottle, a small number of mothers combined breastfeeding and formula supplementation, whereas for others, it led to the complete discontinuation of breastfeeding. This mother (Participant 5) explained, “Once I supplemented with the formula, I took advantage of bottle feeding and I slowly stopped breastfeeding.”

#### Availability and quality of social support

Adolescent mothers who were encouraged and supported to breastfeed by family members and their partners were more positive about their breastfeeding experiences. To manage breastfeeding difficulties, adolescent mothers identified their family as a primary source of support. Most often this was their mother, stepmother, or another female relative who had previous experience with breastfeeding. These individuals provided support in terms of encouraging the mothers to continue breastfeeding and to overcome breastfeeding challenges.

Breastfeeding practices were influenced when family members or partners expressed negative attitudes towards breastfeeding or spoke positively about the perceived benefits of formula. Adolescent mothers identified their family members as being non-supportive of breastfeeding when that family member personally lacked experience with breastfeeding. When this was the context of the social network, mothers experiencing breastfeeding challenges were encouraged by family members to introduce formula. As one mother (Participant 7) expressed, “everyone told me that breastfeeding was not as good as bottle-feeding.” Another mother (Participant 2) explained, “My stepmom because she formula fed all of her kids and … as soon as I gave her formula she started growing.”

The mothers expressed that formal support, most often from nurses in the early postpartum period, was beneficial for increasing their knowledge, skill and confidence in breastfeeding. Mothers particularly valued the information and hands-on assistance they received in hospital, as one participant (3) shared:

"It helped me a lot, I felt encouraged and it helped me with the actual how to do it properly, like how to open his mouth and tickle his cheek and break the latch. You know, like how to actually do it…What I really found helpful was the nurse was telling me that he was learning just like I was. Which encouraged me and I felt good about that… it encouraged me to keep trying."

Furthermore, the hands-on assistance and encouragement the mothers received while in hospital reinforced the information about breastfeeding that was received in the pre-natal period. This is evidenced by this mother’s experience: “I wasn’t sure of what I was doing, even though I had that information [prenatally] … you need the physical part to actually show you how to do it, that’s why I liked it at the hospital” (Participant 12). Once discharged from hospital, the participants rarely described actively accessing professionals for support.

While family was the primary source of breastfeeding support and information identified by the mothers, peer support also had an impact on breastfeeding practices. Being in a supportive environment surrounded by other young mothers was identified as influencing breastfeeding experiences. As one mother (Participant 1) in a school program for young mothers explained:

"Not all the girls here breastfeed, only like a couple of them, but when I saw them they were breastfeeding, whatever. I didn’t feel so singled out. I could just sit there and I could just talk to other people even while feeding so it wasn’t like concentrating on, ‘Ok, now we gotta breastfeed’ and we were just relaxing, all of us while our kids were eating. So it was a lot more comfortable and I was good after that."

When asked about accessing professional breastfeeding support, another adolescent mother (Participant 1) explained, “I didn’t feel I really needed to, I had enough support [through family/friends].” Interestingly, the vast majority of adolescent mothers were aware of the community-based breastfeeding services available, but when faced with barriers to breastfeeding, did not feel the need to access them.

#### Mothers’ knowledge of breastfeeding practices and benefits

A significant number of participants held beliefs that contradicted current evidence and existing best practice breastfeeding guidelines including information about the process of milk production and supply, quality of breastmilk, and normal infant feeding patterns. One mother (Participant 11) illustrated this by stating,

"Because I didn’t know, even if that was normal, I didn’t know that. I didn’t know the pain was normal, I didn’t know that, I thought milk just came out at first and I just, I didn’t know anything…I thought it was just so much easier than that. I didn’t think there’d be any problem. It totally surprised me; I thought it would be so much easier."

Mothers held unrealistic expectations about the ease and process of breastfeeding and many were surprised to experience pain or challenges with latching.

In addition to concerns regarding frequency of feedings and having adequate milk production, adolescent mothers disclosed concerns regarding their own nutritional intake and its impact on the quality of their breastmilk. The following quote demonstrates how concerns for maternal food consumption impacted on the decision to continue to breastfeed:

"I don’t make a whole lot of money and I can’t always buy good food and stuff so it was hard for me to actually eat and then breastfeed and provide enough for her…so that’s one of the reasons why I stopped. (Participant 6)."

Although some mothers identified an inability to afford nutritious food, they did not identify the economic benefits of breastfeeding over formula feeding as rationale for increased breastfeeding duration. However, the mothers were able to identify the cost savings of breastfeeding, quoting “It is cheaper…the money you save on breastfeeding is unbelievable” (Participant 5).

However, a small number of the participants shared their perceptions about the positive benefits of breastfeeding, particularly with respect to infant health. In their discussions about breastfeeding these mothers perceived that breastmilk was the “best” source of nutrition for their infant and that by feeding on demand, their infants were getting the “exact” amount of nutrition they needed.

#### Mothers’ perceived sense of comfort in breastfeeding

The participants spoke extensively about how their experiences of being young mothers influenced their breastfeeding practices. Many mothers described feeling uncomfortable breastfeeding when they were in front of other people or out in public. Some mothers discussed breastfeeding in front of family members and identified they were often limited in finding a private space to feed, including in their own home. A mother (Participant 1) describes her experience feeding at home:

"…in the hospital I was closed in I was all by myself, so it wasn’t [embarrassing] … I had more privacy when I was in the hospital then I did when I was at home… so many people were living here [at home] all the time, it was just oh my gosh I had no privacy… they’d walk right in my room."

Breastfeeding in public was a very specific context in which the adolescent mothers described feeling anxiety, embarrassment and a fear of being judged. In the mothers’ narratives, we interpreted that they perceived others judge them for being young and pregnant and as such, not meeting societal expectations of them. In describing the experience of breastfeeding in public, one mother (Participant 1) shared “I know they were talking about me because it’s something you know if they’re obviously looking at you, pointing at you, they were staring at me too.” Several of the adolescent mothers echoed this sentiment and described feelings of being watched and judged. In continued discussions, several mothers identified seeking out secluded places to breastfeed their infant whereas others managed their feelings of discomfort by feeding their infants with a bottle. In contrast, when participants had the opportunity to be within a peer group of breastfeeding young mothers, they did not feel that they were being judged and as a consequence, felt more confident in their ability to breastfeed in public.

## Discussion

In this study, we explored the breastfeeding experiences of a cohort of adolescent mothers in Durham Region, Ontario, Canada. It allowed for identification of barriers and supports from both formal and informal sources based on the unique experiences of adolescent mothers. While many of the experiences of the adolescents were similar to those of adult mothers, this study provides valuable insights into specific factors that influence breastfeeding initiation and continuation in this population. These can be enabling factors if they are present and positive, or can work to negatively affect the breastfeeding experience and act as barriers to breastfeeding.

Adolescents’ infant feeding decisions and experiences are influenced by the unique challenges related to this specific stage of life. Similar to the results of this research, previous research has found that adolescents’ perceptions regarding public breastfeeding, breastfeeding support from the adolescents’ mother, boyfriend, and friends, and breastfeeding knowledge, significantly affect breastfeeding decisions and outcomes [10,14,17,18]. It is at the critical stages of adolescence when self concept, role attainment, decision making skills, among many other developmental tasks are achieved [33,34]. The developmental stage that an adolescent mother is at will impact her perceptions of herself as a mother. Likewise, being a mother will also influence the perception of self as an adolescent.

When an adolescent becomes pregnant and subsequently begins to parent her infant, the tasks of adolescent development must be balanced with that of the role of becoming a mother [10]. Adolescence is a bridging point between childhood and adulthood, whereby the adolescent must figure out her role identity, define her values and discover her role amongst peers to progress into successful adulthood [24,33,34]. This was identified in this study as adolescent mothers described their competing roles; the draw of social life, friends and partners versus their responsibilities as a new parent, breastfeeding demands and time commitments.

The current study revealed that adolescent mothers’ prenatal expectations to “try” to breastfeed appeared to be met by any single attempt to breastfeed. Swanson’s examination of planned behavior theory found that participants’ own perceptions of societal norms impact their choice of infant feeding method [35]. Within this theory, adolescents have a varying context from adult mothers of what is deemed socially acceptable. Societal norms within the adolescent context is to seek if breastfeeding fits into their life circumstances and norms. The participants’ actions to simply attempt to breastfeed did not mean that they were committed to continued breastfeeding for any extension of time. It appeared that when the expectation to attempt to breastfeed was the goal, it was met whether or not the mother continued breastfeeding. Similarly, an American study consisting of 24 focus groups found that prenatal participants spoke of their commitment to “try” to breastfeed rather than to “learn” to breastfeed [22]. The same study identified formula feeding mothers made their decision to stop breastfeeding when they found breastfeeding did not meet their expectations of being “easy” or “natural” [22]. Previous research results are also consistent with the finding that adolescent mothers’ level of commitment to breastfeeding is a significant factor during the process of establishing and continued breastfeeding [10].

Adolescent mothers in this study lacked knowledge about breastfeeding such as how frequently babies feed and how to know if the baby was getting enough breastmilk. As well, some of the participants identified concerns that their own nutritional intake may affect the quality of their breastmilk. Lack of practical knowledge in breastfeeding such as knowing signs of satiety can lead to frustration and resultant early supplementation. In a survey of 53 mothers (14–19 years) to examine adolescent breastfeeding experiences and related behaviors after their postpartum stay in hospital, Spear [20] similarly found that many mothers reported not receiving adequate information about the health benefits of breastmilk for infants and the positive maternal outcomes associated with breastfeeding. Utilizing House’s (1981) social support theoretical framework as cited in Grassley [36], instrumental and informational support are noted as a means by which nurses can support the adolescent. Instrumental support within this literature review is defined by the “practical and tangible” (p. 718) aspects; whereas informational is self evident in providing current, relevant and consistent information such as dispelling of breastfeeding myths, or signs of breastfeeding going well [36]. This highlights the need for health care providers to offer both prenatal and postnatal health education which includes assessment of mothers’ practical breastfeeding skills and perceptions including health education encompassing effective infant feeding, signs of satiety, breastmilk quality, and nutrition [37]. In the early postpartum period, both in hospital and during home visits, nurses have opportunities to provide esteem support and bolster confidence by providing affirmation and encouraging feedback as the adolescent learns to breastfeed [36]. Mothers in the current study valued the support they received from nurses through encouragement, information and practical hands-on instrumental support. They verbalized that such support increased their confidence to breastfeed their baby, which substantiates Grassley’s [36] conclusions that esteem support can build breastfeeding confidence. This also highlights a developmental difference between adolescent and adult mothers. Although all mothers benefit from social support, the developmental stage of adolescence leads to seeking external sources of affirmation as a means to reinforce identity and formulate new roles [33,36]. Whereas developmentally, adult mothers have already formulated their unique identity and rely less on external reinforcement of roles [33].

Breastfeeding in public and breast exposure has been perceived as a barrier to breastfeeding for adolescent mothers [19]. Issues about body image and privacy have been cited in literature as posing a barrier to adolescents’ breastfeeding in public [14]. In the current study, this did not emerge organically from the participants’ discussion about their decisions to initiate or continue breastfeeding. Many of the mothers verbalized feeling comfortable to breastfeed in public settings, however still chose to seek out secluded methods to feed. Unlike the findings from Dykes et al. [19], adolescents in the present study did not identify embarrassment of showing their breasts as the motivator to avoid public breastfeeding. Rather, participants verbalized feeling watched, both for being a young mother and for breastfeeding in public.

Additional barriers are faced by adolescent mothers beyond those faced by adult mothers. Both share the challenge of societal stigma associated with breast exposure for the purposes of breastfeeding in public. However, the additional stigma ascribed to being a “teen mother” further complicates the breastfeeding experience for the adolescent mother. In a structured review of the qualitative literature synthesizing adolescents’ experiences of breastfeeding MacGregor and Hughes [38] identified the issues of being judged and feeling embarrassed as consistent barriers, identified across multiple studies, that contribute to mothers’ low confidence in feeding in front of other individuals. Even more significant, in our study, participants indicated that their experiences of feeling judged strongly influenced their decisions to quit breastfeeding altogether. The negative consequences of stigma surrounding adolescent pregnancy have been well documented in multiple contexts [39,40], demonstrating the need for public education in being more supportive of breastfeeding and acceptance of young mothers as equal participants in the parenting role as their older counterparts.

The positive or negative support received from adolescents’ social system interacts with barriers to breastfeeding. In looking at social supports, if it was deemed present and positive, it was identified as being supportive of breastfeeding. If the social support was negative to breastfeeding, it was perceived by adolescent mothers as a barrier to breastfeeding. In a literature review, family support is noted as an important aspect of breastfeeding support for the adolescent, particularly in terms of emotional support [36]. Emotional support entails providing empathy, building trust and concern surrounding the adolescent mothers’ breastfeeding experience [36]. Furthermore, it was found that family, in the form of the adolescent’s own mother and the father of the baby, served as a form of network support; essential to the longevity of breastfeeding and overcoming breastfeeding obstacles. It is within this positive, supportive informal social network that the adolescent receives encouragement, emotional and confidence-building support [36]. Family support has played an important role in adolescent breastfeeding experiences in previous research [19]. In findings from Mossman et al. [14], more mothers initiating breastfeeding had partners who were positive, or supportive of the decision to breastfeed. When mother-figures of the adolescent participants in the current study were positive towards breastfeeding, adolescent mothers identified feeling supported in their breastfeeding experiences. Within the theoretical social support framework, this form of esteem or appraisal support serves to encourage the adolescent, build on her confidence and to verify her independence and decision making [36]. When support was identified by the current study participants as negative, such as the her own mother-figure being unsuccessful in breastfeeding, this was identified as a barrier to the participants’ breastfeeding experience.

Most notably, when faced with breastfeeding challenges, adolescent mothers did not identify a need to receive professional help and therefore did not actively access professional support. Obtaining information from books, pamphlets, or online was rarely mentioned. When looking for help, it was sought from interpersonal sources such as family or partners. Mothers perceived that they had enough support through their informal networks and therefore did not identify a need to seek other support. They also discussed their peers as positively influencing their breastfeeding experience. Their descriptions are congruent with previous literature examining the support needs of adolescents; concluding that breastfeeding peer networks were an invaluable aspect of the support spectrum for these mothers. The nurse provides network support by linking mothers to their informal sources of support, including peers with breastfeeding experience [36]. Previous studies have also found that informal breastfeeding social support was highly valued to adolescent mothers [10,14,36]. The adolescent mothers in this current study identified their family and partners as their primary source of support. At this stage of development, adolescents’ autonomy and decision-making abilities are still developing and they are seeking direction from their social supports [24]. They may still have a need to be mothered and supported, while figuring out their own roles as individuals and as mothers.

### Professional implications

When thinking about strategies to promote and support breastfeeding among adolescent mothers, this study provides some valuable insights. The narratives of adolescent mothers offer a rich source of information for health care professionals to develop an understanding of the barriers and supportive factors that impact breastfeeding. Adolescents are diverse in their developmental stages and their abilities to comprehend and respond to specific tasks and expectations [41]. Developmental theory and stage of development provides context in which health care professionals must consider when working with the adolescent population. Recognition of determinants of health, including the socioeconomic context experienced by the adolescents, must also be considered when looking at health promotion strategies [42].

A significant finding that came from this study is that adolescent mothers often do not identify themselves as needing professional support or interventions. Therefore, active engagement of such clients is required in order to be supportive of breastfeeding practices in the adolescent population. Active engagement of adolescents was noted in a literature review, citing adolescents as being hesitant to ask for information [36]. The current study provides insight into this phenomenon through the narratives of participants expressing they did not recognize themselves as needing support and instead looked to informal networks for breastfeeding assistance and information. Additionally, many of the adolescent mothers identified making their decision to breastfeed prenatally. Therefore, health care professionals need to make the timing of their interventions accordingly to establish rapport, address knowledge deficits and collaborate in establishing breastfeeding goals. Existing evidence supports this finding and goes further to recommend prenatal assessment of breastfeeding attitudes and myths [14].

As well as providing prenatal breastfeeding support, health care professionals need to consider early postnatal intervention. This finding is congruent with the recommendations of a previous study citing the need for more long-term follow up for young mothers [20]. Demonstrating the importance of intervening both pre- and postnatally, Wambach et al. [43], conducted a randomized controlled trial to evaluate an education and counseling intervention designed to promote breastfeeding initiation and continuation for adolescent mothers (15–18 years) starting in the second trimester and continuing until the fourth week postpartum. The intervention, led by a lactation consultant and a peer counselor, was successful in influencing breastfeeding duration, but not rates of initiation or exclusive breastfeeding. Consideration should be given to providing adolescent mothers an early postpartum home visit regardless of whether breastfeeding problems have been identified by the client or professional. This early intervention will allow for assessment of potential barriers that arise before breastfeeding is fully established. As well, careful assessment and linking of the adolescent to breastfeeding peers can be a network support that the nurse is situated to provide both in hospital and within the community. Moreover, positive reinforcement of the adolescent’s current success, confidence building, and reinforcement of practical aspects of prenatal breastfeeding education can occur during this time. Such interactions will also help foster a relationship with a professional and will provide an opportunity for network support and connection to targeted community services. Furthermore, it will be an opportunity for encouraging the adolescent mother to access these services and increase recognition that the services are there for her as well as her adult counterparts. This is the interpersonal connection needed to identify the professional as another source of support.

For this group of adolescents mothers who are demonstrating low levels of motivation to initiate or continue to breastfeed or who are experiencing some level of ambivalence about the process, the counseling strategy of motivational interviewing may show promise as a tailored intervention to integrate into prenatal education services and early postpartum home visits to support this population in initiating and continuing to breastfeed. Motivational interviewing is a counseling strategy employed by clinicians to engage with clients in exploring and resolving feelings of ambivalence related to behavior change [44]. In a meta-analysis of 119 studies that evaluated the effect of motivational interviewing compared to controls or other interventions, across a range of targeted outcomes or health related-behaviors, Lundahl et al. [45] concluded that the use of motivational interviewing tends to produce statistically significant effects across different contexts.

Although there have been no evaluations of the effectiveness of motivational interviewing with this specific population for the purpose of addressing ambivalence and behavior changes with respect to breastfeeding, the intervention shows potential for adoption and evaluation in public health nursing practice. In a qualitative study, Racine et al. [46] interviewed 44 low-income breastfeeding women to identify incentives and disincentives to breastfeeding within the first six months of the postpartum period. Within the sample, women were classified into three groups: intrinsically motivated, extrinsically motivated (where breastfeeding decisions were influenced by other people), and successfully experienced (a woman who had breastfed successfully with past infants and who was intrinsically motivated). Extrinsically motivated women were on average younger, breastfed for a shorter duration of time and were less likely to be breastfeeding at six months compared to women in the other two categories. They recommended the need to tailor interventions specifically to women’s level of motivation and identified motivational interviewing as a strategy that might demonstrate success in promoting breastfeeding initiation and continuation among extrinsically motivated women. Wilhelm et al. [47] conducted an experimental two-group design to explore the feasibility of using motivational interviewing with primiparous mothers (19–38 years) to promote sustained breastfeeding. While the results were not statistically significant, a sample of 73 mothers who received motivational interviewing as an intervention, breastfed for 98 days compared to those who were in the control group at 81 days [47].

Another area that can be influenced is the confidence of adolescents. General confidence building and self esteem strategies for adolescents may be beneficial [14]. Confidence building can be achieved through engaging informal supports such as the adolescent’s mother or intimate partner in the instrumental and informational aspects of breastfeeding support that the nurse provides [36]. Furthermore, the emotional support provided by family helps the adolescent mother feel supported and cared for, creating an environment conducive to achievement of new learning. As well the nurse and family can provide esteem support that will enhance the adolescent’s confidence to breastfeed by providing encouragement and a sense of empowerment over her new role [36]. Increasing women’s confidence in their ability to breastfeed, both prenatally and continuing into the postpartum period, may aid in increasing commitment to breastfeeding. It was found that women with low confidence in breastfeeding are more likely to stop within the first week after birth [26]. Health care professionals have multiple opportunities to enhance their efforts to include adolescent mothers’ social support people in the provision of pre- and postnatal care, as they are most often the primary source of support [14]. Negative social support, such as the adolescent’s own mother-figure, can influence the breastfeeding experience. Therefore, professional support can also be directed at changing attitudes and beliefs of social support individuals regarding breastfeeding. At a population level, advocacy efforts should be directed to support breastfeeding in public, reduce the stigma surrounding adolescent parenting and create a culture of acceptance to breastfeeding as the norm for all mothers.

### Study strengths and limitations

This descriptive qualitative study was the first to explore and describe adolescents’ breastfeeding experiences in this Canadian region. The recruitment of a fairly homogenous purposeful sample of mothers allowed for the quick emergence of saturation among study themes. Despite the small sample size, strategies to promote overall data credibility and dependability were integrated into the study design including researcher triangulation, peer examination and the use of multiple coders to review and interpret the data. However, while a strategy of purposeful sampling was used to identify participants with specific characteristics, all mothers meeting the inclusion criteria were invited to participate. The use of this type of purposeful, convenience sampling limits our understanding of the full scope of experiences among adolescent mothers. It is important to note that the adolescent mothers in this study were not looked at in terms of ethnic diversity however findings from this study are consistent with results from studies examining the same phenomenon in different ethnic populations of adolescent mothers or geographic regions of North America. Within this study there are also some notable limitations that may influence transferability of the findings to other contexts. All the participants in this study include predominantly primiparae mothers, and therefore did not look into the impact of previous births and breastfeeding experiences as positive or negative factors. The study did not include any participants from more rural areas of the region studied, nor did it include participants younger than the age of sixteen. Based on these limitations, the findings of the study should be interpreted with caution if they are to be applied to other populations. It is recommended that future research include mothers younger than sixteen, as well as including cultural breastfeeding practices and their impact on the breastfeeding experience of the adolescent mother.

## Conclusion

This study has contributed to the body of evidence on adolescents’ experiences of breastfeeding, and is one of the few studies to document this phenomenon within the Canadian context. This qualitative evidence provides health care providers new conceptual insight and understanding of the factors that influence adolescents’ decisions to “try” breastfeeding and to continue providing breastmilk to their infants.

It is important that adolescents have the necessary information to make fully informed decisions, and the positive social networks to support their breastfeeding experience. Without these supports, they may experience ambivalence in their decision-making about breastfeeding, creating opportunities for health care providers to tailor interventions specifically for this population. Future interventions should be theoretically informed, build upon a therapeutic nurse-client relationship, start in the prenatal period, extend into the early postpartum period, and include the integration of motivational interviewing.

## Competing interests

The authors declare that they have no competing interests.

## Authors’ contributions

HR and KP conceived of study. HR, KP and JCB participated in study design and in methodological and ethical review. HR and KP conducted participant interviews. All authors participated in data analysis and interpretation. HR wrote report. SAN and KAC conducted literature reviews, literature synthesis, participated in ethical review of peer debriefing process. KAC conducted peer debriefing. SAN, KAC and SMJ wrote the manuscript. All authors read and approved of the final manuscript.

## Pre-publication history

The pre-publication history for this paper can be accessed here:

http://www.biomedcentral.com/1471-2393/12/149/prepub
